# Measuring the Reliability of Picture Story Exercises like the TAT

**DOI:** 10.1371/journal.pone.0079450

**Published:** 2013-11-05

**Authors:** Nicole Gruber, Ludwig Kreuzpointner

**Affiliations:** Department of Psychology, Universität Regensburg, Regensburg, Germany; University of Rochester, United States of America

## Abstract

As frequently reported, psychometric assessments on Picture Story Exercises, especially variations of the Thematic Apperception Test, mostly reveal inadequate scores for internal consistency. We demonstrate that the reason for this apparent shortcoming is not caused by the coding system itself but from the incorrect use of internal consistency coefficients, especially Cronbach’s α. This problem could be eliminated by using the category-scores as items instead of the picture-scores. In addition to a theoretical explanation we prove mathematically why the use of category-scores produces an adequate internal consistency estimation and examine our idea empirically with the origin data set of the Thematic Apperception Test by Heckhausen and two additional data sets. We found generally higher values when using the category-scores as items instead of picture-scores. From an empirical and theoretical point of view, the estimated reliability is also superior to each category within a picture as item measuring. When comparing our suggestion with a multifaceted Rasch-model we provide evidence that our procedure better fits the underlying principles of PSE.

## Introduction

Many psychological constructs cannot be measured directly. In the classical test theory (e.g. [1]) each observed score is decomposed into a true and error score. To examine the reliability of a test —one of the central criterions of its goodness— many methods were developed. If a test measures a time-stable construct, the score achieved in the first session should not differ from the score in the second session (retest reliability). If a test contains items all measuring the same construct, these items should be highly statistically related (esp. method of split-half or internal consistency).

When these general methods of calculating reliability are used for projective tests, they mostly yield unacceptable scores. One well known projective measure is the Thematic Apperception Test (TAT), by McClelland since 1989 mostly called Picture Story Exercise (PSE [2]). Participants view some pictures, each for about half a minute and are then instructed to write a short story about it by answering some leading questions. Within about five minutes they have to respond. The central assumption of a PSE is that participants identify themself with the protagonist of the picture when writing the story and thus project their own needs into their story. Those stories are coded for implicit motives using a special coding system. This coding technique of PSE has been widely used on different versions. Most common is the measure of the need for achievement [3-5]. Heckhausen’s PSE (English language translation by Schultheiss [6]) assessed two components of need for achievement separately: (1) hope of success (HS) and (2) fear of failure (FF). Heckhausen stated this two components as interrelated with each other. He calculated a “net hope score” (NH) as HS - FF and the “resultant achievement motivation” as HS + FF. But recent studies and theories (e.g. the quadripolar model [7]) imply the distinction of the two components.

Even though the achievement TAT is a well-researched and empirically validated assessment, its reliability has often been criticized. For example, Entwisle [8] stated in a review about PSE (or like she called: fantasy-based measures of achievement motivation) that the internal consistency “rarely exceeds .30 to .40” (p. 377), but listed a few results with obvious higher as well as obvious lower values. For retest reliability she found studies with values about .30 and lower. When equivalent forms were used the values were mostly higher (>.50). Lundy [10] observed a loglinear degression of retest reliability for the time between two measurements and concluded for the one day a mean stability coefficient of .71, .60 for a week until .25 for 10 years. They stated that the retest reliability of TAT mostly is in an acceptable range. Schultheiss and Pang [9] also assessed the reliability of two PSE, found retest reliabilities “in the same range as those of these [MMPI, CPI and 16PF] three popular and representative objective personality tests” (p. 143) of .48 and .56, but alphas of .32 and .31 for the first and -.18 and .22 for the second measurement a year later. He conceded (p. 144): “The inevitable conclusion is that the assumptions of classical psychometrics are not met with TAT, and that alpha is therefore an inappropriate measure for this test.” Current researchers (e.g. [11]) have “accepted the unreliability of TAT” ([12], p. 100). But claiming PSE as “test-theory free” because of low reliability scores is no solution, indeed it shows that reliability calculations for projective tests have always been a big problem.

McGrath and Carroll [13] reported in their critical review about PSE low internal consistency and retest stability but an adequate inter-rater reliability. But inter-rater agreement is not a measure of reliability in the context of the classical test theory, it is a prerequisite of reliability because the measure indicates the independence of the results from the persons who scored the results (i.e. objectivity). We focus in this article on the internal consistency of the PSE. Therefore we first review the Coefficient α by Cronbach [14] and the six lambdas of Guttman [15]. Then we introduce a new reliability calculation using the categories instead of the picture-scores. We contrast this measure with calculations on dichotomous item-level data. We also examine whether Rasch-scaling is appropriate for PSE. Finally we demonstrate empirically on three data sets which internal consistency method best fits the Heckhausen PSE.

### Internal consistency

Cronbach [14] emphasised that by only demonstrating whether two halves of a test are consistent with each other, not all possible variations are examined. To assess the consistency of all items, he constructed the α Coefficient, which is one of the most frequently used measures for internal consistency. One possible reason for its wide use could be that historically it was easy to compute and the measure is a perfect fit to self-rating questionnaires with a high number of similar items. However, α could inflate the reliability of a test, especially self-rating scales, because people like to reflect a consistent self-concept [16]. But if the items are not equivalent or even if they are heterogeneous, α can produce misleading reliability scores and therefore should not be used. Rae [17] discussed the problem of α and stated that the assumption for its use “implies that every person’s true score on any given component differs from his or her respective true score on any other component by only an additive constant” (p. 177). Borsboom [18] queried the correct usages of the true score concept in most measurement research at all. When he termed the concept of lower bounds as “probably the most viable defence that could be given for the standard practice in test analysis” (p. 30), he questions a procedure which is in use more than 70 years. Six years before Cronbach [14], Guttman [15] proposed six coefficients for internal coefficients and established the concept of lower bounds. Guttmans λ_3_ is the exact equivalent of Cronbach’s α. Guttman started with λ_1_, which is very similar to λ_3_, but the calculation did not include the number of items. As an improvement, he included the numbers of items as well as the covariances in the λ_2_ coefficient. Additionally he developed as a short version of λ_2_ λ_3_, because it “is easier to compute than λ_2_” (p. 274) by ignoring the covariances. However therefore, two prerequisites for its use are strict homogeneity and positive covariances. In all other cases, Guttman [15] suggested to use λ_2_, despite the increased computational requirement. Three further coefficients were developed: λ_4_ is a measure for split-half reliability for which covariance is not calculated, λ_5_ is another measure developed for the case that one item has “large absolute covariances with the other items compared with the covariances among those items” (p 277). λ_6_ is a measure when data fit to regression model like McClelland [19] assumed for PSE by using the multiple regression error variance instead of the item variances. Although Fleming [20] by referencing Lundy [10] (Fleming cited an unpublished version of 1980) suggested the assessment of the reliability of PSE in using linear regression, a calculation of λ_6_ for a PSE was not findable.

 Revelle and Zinbarg [21] subsume the discussion about the use of several coefficients as lower bound of internal consistency. They recommend to use ω_t_ ([22]), especially in “contexts, such as applied prediction, in which we are concerned with the upper bound of the extent to which a test’s total score can correlate with some other measure and we are not concerned with theoretical understanding regarding which constructs are responsible for that correlation” ([21], p. 152). In case of a unidimensional construct ω_t_ = α. When the goal is to assess “the degree to which the total scores generalize to latent variable common to all test items” (p. 152), the use of *ω*_*h*_ is more appropriate. For PSE all of the above named methods can be applied. But this does not mean that they are all appropriate.

According to the Dynamics of Action Theory (DoA), Atkinson and Birch [23] described the problem when using a PSE that a trait (the motive) can only be measured by the situational state (the motivation) which has been known to fluctuate for several reasons. An inherent response behaviour to the pictures is the lowering of the need for achievement activation force through writing an achievement-thematic story. The need to write about an achievement topic in the next picture (the next item) decreases. Consequently, the progress will be up and down, especially for highly motivated people. McClelland [24] referred to Atkinsons doctoral thesis since then this “so-called sawtooth effect in the achievement content of successive stories has been known” (p. 31), when people do not write the same or similar story just because of the instruction to be creative. Consequently this effect leads to low values of statistical indices of internal consistency. So Atkinson and Birch [23] tested their theoretical assumption with computer simulations and found according to their hypothesis that the high criterion validity of TAT was consistent with very low and even negative reliability scores. Atkinson, Bongort and Price [25] hypothesized that ipsative variability, which is associated with low internal consistency, will increase the criterion validity (assessed with an arithmetic task) of the motivational imaginary story. The results revealed an outlandish internal consistency of -1.23 (assessed with Coefficient α) referred to a good criterion validity of .62. Reumann [26] suggested that calculating internal consistency using α would not be effective, because he expected this measure in a well-constructed PSE to become infinitely negative.

Tuerlinckx et al. [11] also tested the theoretical assumptions of Atkinson and Birch [23], but could not validate them. They found that some pictures stimulate a high achievement motive and some do not but no evidence was found to explain why. Thus, the result best fits a model of spontaneous-drop-out, which was later theoretical explained by Schultheiss et al. [27] using the Cognitive Affective System Theory (CAST; [28]). This theory offers an explanation for finding which disagree with the drive reduction suggested by the DoA theory and is very similar to the explanation provided by McClelland [24]: People learn to satisfy their needs in different situations throughout their life. So some people think of an instructor and worker when they see two men standing on a workbench, others think of father and son or two friends drinking beer. This suggests that each score results from an interaction between the picture-cue and the personal background of a person which cannot be controlled (another reason for fluctuation).

This unpredictable change of item difficulty is an immense problem for the calculation of the reliability, because all unpredictable changes serve as measurement error. Another problem is that not all pictures correlate highly and positively with each other. Every picture can stimulate the motive in a different way, which leads to completely different stories to the extent that they correlate negatively. Moreover, α increases with the number of items, but PSE comprises few items, because people get tired after more than six pictures ([19]).

In sum, we state that α is not an appropriate reliability coefficient when using the sum of occurring categories of each picture as items of a PSE, because the items (picture-scores) are inhomogeneous. We provide another approach of calculation to eliminating the inhomogeneity.

### Category vs. picture reliability

Therefore, we introduce an idea that eliminates the inhomogeneity by taking a closer look at the internal consistency of the coding system. The scores of pictures are always related to the underlying coding system, but after thorough review of the literature the reliability of a coding system has not been assessed in any study according to the reliability of the projective test. The only exception is Kuhl [29], who assessed reliability in the context of Rasch-scaling methods, and Lundy [10] by mention the possibility to use the scores of categories for regression equations. Our idea is to use the categories instead of the pictures as items. For example, when calculating the reliability of the *hope of success* scale, the scores for each of the six pictures are not used but the scores of the six categories. Each item consists of the number of pictures which fits the criterions of the category. The overall participant score remains the same. Generally, we assume that calculating reliability using categories instead of pictures as corresponding items would be a much more adequate measure for internal consistency of PSE. The categories of the coding system are constructed to correlate positively and to be homogeneous. Participants with a high need for achievement are expected to write more elements which fit the criteria of the categories. Though the influence of the length of the stories of a subject, which affected the motivescore - e.g. Pang and Schultheiss [30] found a correlation of .23 -, has less impact for the estimation of the reliability. Thus, the relevance of the saw-tooth-effect according to the DoA or the picture cue effects as specified in the CAST will be minimized. To shortly explain this with an example data-matrix (see table 1).

**Table 1:** Example data matrix for seven subjects with sums of categories (cat1, cat2, cat3) and sums of pictures (A, B, C).

	Picture A			Picture B			Picture C									
subject	Cat 1	Cat 2	Cat 3	Cat 1	Cat 2	Cat 3	Cat 1	Cat 2	Cat 3	Sum	Cat 1	Cat 2	Cat 3	A	B	C
1	1	1	1	1	1	1	1	1	1	9	3	3	3	3	3	3
2	0	1	1	1	0	1	1	1	0	6	2	2	2	2	2	2
3	0	0	1	1	0	0	0	1	0	3	1	1	1	1	1	1
4	0	0	0	1	1	1	1	1	1	6	2	2	2	0	3	3
5	1	1	1	0	0	0	1	1	1	6	2	2	2	3	0	3
6	1	1	1	1	1	1	0	0	0	6	2	2	2	3	3	0
7	1	1	0	1	0	1	1	1	1	7	3	2	2	2	2	3

This is a very constructed and shorten data-matrix of a PSE data set. We just use three categories (Cat 1, Cat 2 and Cat 3) and three pictures (A, B, C). This way the data of PSE can be seen as a two-level matrix consisting of 0 and 1, whereby categories are nested within the pictures. As for the first three subjects the sum of pictures and the sum of categories are equal, for the second three subjects the equal scores within the three categories leads to different scores for the pictures. So there are high intercorrelations for the categories and low intercorrelations for the picture-scores (see table 2).

**Table 2:** Intercorrelations of categories (cat1, cat2, cat3) and pictures (A, B, C).

	Cat 1	Cat 2	Cat 3			A	B	C
Cat 1	1.00				A	1.00		
Cat 2	.84	1.00			B	-.13	1.00	
Cat 3	.84	1.00	1.00		C	-.12	-.12	1.00

This statement is also checked mathematically by reviewing the formulas provided below. Strongly simplified, the internal consistency measured with α can be seen as a relationship of test variance (V_t_) and item variance (V_i_) ([14], p. 304 (13)):

$$\alpha =\frac{n}{n-1}\left(1-\frac{\sum V_{i}}{V_{T}}\right)$$ (1)

*Note: i* is counter of *n* items.

An obvious feature of equation 1 is that regardless of using categories or picture-scores, the denominator will be the same because the test variance is the same in both cases. Hence, there are only two reasons why reliability calculated over categories would be higher than reliability calculated over pictures. First, the item variance for categories is lower than for pictures, which makes sense if we follow the assumptions of Atkinson and Birch [23] or of Schultheiss et al. [28] that each picture stimulates the motive to varying extents. But all categories are always related to the same criteria, thus the variance of categories should be lower. Second, higher covariances are expected when using category-scores instead of picture-scores. If the coding system is valid, all categories should positively correlate. We can neither hypothesize it for pictures (e.g. DoA) nor observe it (e.g. [26]).

Given the denominator, the difference between these two types of reliability measures depends on the numerators. We found a direct connection of internal consistency calculated from pictures and from categories. We first decomposed the variances of the internal consistency numerator calculated over pictures. The variance of a sum is the sum of the summand variances and each summand pair covariance (e.g. [31], p. 72, (4.32)). To calculate variance and covariance, we can use equation 22 and 24 by [15] p. 269: (*σ*_*sj*_)² = *E*(*x*_*ijk*_ *-μ*)² and *γ*_*xgxj*_ = *E*(*x*_*igk*_-μ_*g*_)(*x*_*igk*_-μ_*j*_) respective, where E is the expected value that can be estimated as sum of all components divided by *n*.

To simplify and because the actual measure of variability matters little for our suggestions, we preferred the sum of squares (SS) instead of the variance of the picture-scores (*Var*_*p*_):


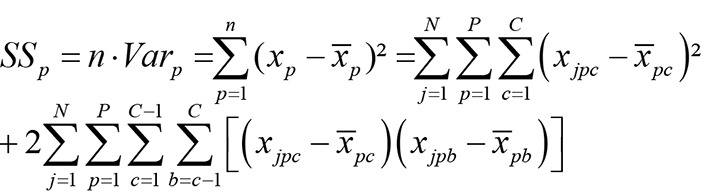
(2)

*Note: j* is counter of N subjects, *p* is counter of P pictures, *c* is counter of C categories.

The picture SS is the sum of all category SS by picture-scores and the sum of all sums of products of each category pair mean deviation within each picture. Thus, for example, when calculating the SS of picture A (resp. *p* = 1) each category SS, starting from A1 (category 1) to A6 (category 6) and the sum of products of each category pair (A1, A2) to (A5, A6) are summed. See Appendix S1 for a detailed mathematical proof of this decomposition.

We conclude from this computation that the picture-scores variances depend on the sum of their sub-variances (the variances of all item points x_pc_) and on the sum of their covariances. But this sum of covariance will be high when categorical-covariance is generally high, which in turn leads to high categorical reliability (as can be seen with equation 1).

Reconsidering that the sum of all pair covariances is the total-test-variance minus their variances leads to the following equation for the covariance of the picture-scores:


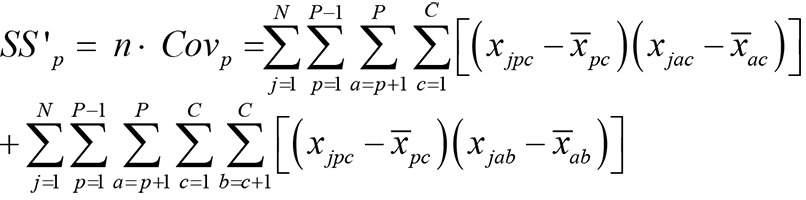
(3a)

*Note: j* is counter of N subjects, *g* is counter of P pictures, *c* is counter of C categories.

and also to this equation for the covariance of category-scores:


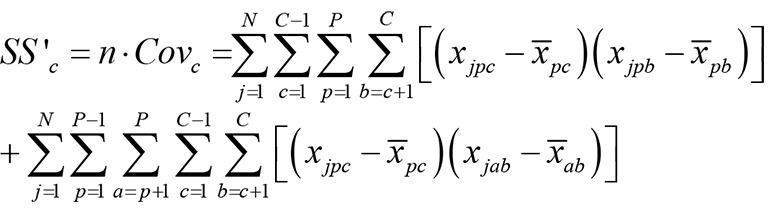
(3b)

*Note: j* is counter of N subjects, *g* is counter of P pictures, *c* is counter of C categories.

See the Appendix S1 and especially Figure S1 and Figure S2 for a detailed explanation.

Equations 3a and 3b differ only in the first term. Thus, if reliability of categories is different from the reliability of pictures, the difference issue from the first term in both equations. But this term is a component of the variance of the other one. For example, the first term in equation 3b (covariance of categories) is similar to the second term in equation 2 (variance of pictures). This finding can be a solution for the main reliability problem and the validity-reliability dilemma of projective tests. The higher the variance of the picture-scores the higher are the covariance of the categories score and the higher are the difference of the α coefficients calculated with pictures vs. calculated with category-scores (see equation S1 in Appendix S1). This indicates that the internal consistency of a valid PSE, if it was calculated with category-scores, would ever be higher as the internal consistency calculated with picture-scores. In sum, calculating reliability using categories instead of pictures would be an optimum solution. In the next step, we contrast this new method with other ideas such as using dichotomous measures [33] or using item response theory for calculating reliability [12].

### Measuring internal consistency of PSE as dichotomous data

Before calculating internal consistency using α, Kuder-Richardson Formula 20 (KR‑20) was a common reliability measure used on each dichotomous single scoring unit [32]. Jensen [33] wrote about KR-20 that “still it is probably the best estimate possible of the internal consistency reliability of the TAT” (p. 123). Currently, support of this type of thinking is lacking, yet it is worthy of discussion. The negative interaction of reliability calculated using the picture-scores will be deleted by breaking down the items to the least possible scoring unit, which leads to a matrix only consisting of 0 and 1.

The advantage of calculating the internal consistency of a PSE using the categories for each picture as dichotomous items is that the value of the reliability coefficient is increased because of the higher number of items. We assume that other factors also contribute to the increase, such as higher homogeneity as a result of the high inter-correlations of the underlying categories, and also their high correlations with the overall score is an important factor [33]. Thus, calculating reliability on a dichotomous level will always be influenced by internal picture and categorical consistency such that high categories-as-items reliability stands in relation with low pictures-as-items reliability (see equation S1). Therefore, we can follow that in a well-constructed PSE the yield score for reliability will always be confounded by the low interaction of picture-scores. Hence, we suppose that reliability calculated on a dichotomous level is still influenced by the inhomogeneity of the picture-scores.

We consider that all measures on a dichotomous level are influenced by the individual style of crossing motive (e.g., someone likes to write more feelings, another one likes to write more instrumental activity) and on the picture which evokes the motive (e.g., someone writes a motive consisting of a story on picture one and not on picture two). But measures on a dichotomous level are based on the assumption that all items are positively correlated with each other.

With PSE generally needs are measured, but the expression of these needs could change during test situation. As it will be shown in our investigation, calculating on a dichotomous level is also influenced by these effects, but when calculating internal consistency using categories as items the problem can be solved. The scores of the categorical system are independent of the picture from which they come. If for example a person’s respond fits the category “instrumental activity” for hope of success in picture A and another person’s respond was influenced the same way but at picture C, because each picture reflects the individual life story of the respective reader (CAST), the categories score will not differ in consequence of that. Likewise the saw-tooth-effect assumed in the DoA theory, when the drive of writing achievement related statements is satisfied in picture B but perhaps high when writing stories for pictures A and C, will be under control when using the categories score for calculating reliability. Here it does not matter which picture story fits the criteria. So, in our opinion, the calculation of internal consistency using categories should be preferred.

### Rasch-Model for higher reliability

As the last analysis, we assessed whether the assumptions of Rasch-modelling have an advantage for the estimation of PSE reliability. Tuerlinckx et al. [11] discussed the possibility of subjecting PSE to Rasch-scaling, assuming that the tendency of giving an achievement relevant answer on each picture (scored with 0 or 1) depends on the strength of the motive of a person and the instigating force of the picture. After testing many Rasch-models, they concluded that PSE best fits a spontaneous drop out model for which some pictures force motive and some do not. Thus, the drop out hindered reliability, which in their opinion could only be solved when increasing the numbers of pictures — an option that they rejected because of practical reasons.

Likewise, Blankenship et al. [12] found the solution of the reliability problem in using a Multifaceted Rasch Model, which is able to control confounders like the influence of the coder. Blankenship and colleagues tried to improve the test and its reliability by identifying new pictures for a better model fit and higher reliability scores. They found Cronbach’s α of .78, .70 and .69 and a Person Separation Reliability (PSR), which is a Rasch-equivalent of α or KR‑20 as stated by Linacre [34], between .24, .56 and .75, but they found a heterogenic result. Consistent with our theoretical suggestions, we agree with [31] and reject the notion that Rasch-modeling would be the best method for measuring the reliability of projective tests, because the theory and problems underlying these tests do not fit with the assumptions of local stochastic independence of the Rasch-model. Only because “the scoring criteria were […] applied independently to each PSE” ([12], p. 101), this method does not suggest that the items (i.e., the pictures) have no relationship to each other. We turn again to the DoA: If a high motive were to be stimulated by the first picture, the answer for the second picture could be based on a lower strength of the need. According to the CAST, this effect of different scores in different pictures is contingent not on the order of the pictures and a reduction of the motive drive but on their content and the interaction with the subject biography. An additional criticism of the Rasch-model is that each picture is seen as one item ([11,12]), which we have shown to be the least optimum basis for measuring internal consistency. This procedure is particularly problematic when, for example, Tuerlinckx and colleagues used pictures only scored as 1 or 0. Such a procedure is not consistent with the theoretical conception of McClelland et al. [4] or Heckhausen [3], who developed this assessment.

### Expectations

Based on the arguments and the procedure that we proposed, we can formulate the following two expectations:

• Measuring reliability using category-scores will outperform methods using picture-scores as items. We should find support for this preference, because category-scores are not hindered by effects of the DoA or CAST as are picture-scores.
• Measuring reliability using categories will also be higher than measuring on the dichotomous level. Measuring on the dichotomous level is influenced by both category-scores and picture-scores. Therefore, the saw-tooth-effect and/or the picture-cue-effect are expected to influence this type of measure.

## Methods

### Participants

We tested our hypothesis first with the data set of N = 35 PSE given by Heckhausen [3] presented in his coding-manual [Data Set S1], because we assume them to be most valid. Second, we used the PSE of N = 113 university students (67 female; age range 19 to 42 years; M = 23.60, SD = 3.00) [Data Set S3]. Additionally, we were able to use the data set of [35] with N = 241 pupils of a vocational school (103 female, age range 15 to 23 years, M = 17.65, SD = 1.63) [Data Set S2].

### Materials

For our investigation we used the PSE of Heckhausen [3]. Heckhausen used six pictures describing a smiling man at the desktop (picture A), a man in front of the directors room (B), two men on a workbench (C), a pupil on a blackboard (D), a man at a desktop (E), two men on a machine (F), whereby three of them mainly activate hope of success (A, C, E) and three activate fear of failure (B, D, F). After having a look at the picture for 20 seconds, the subjects were instructed to answers the four questions: 1. What is going on? Who are the people? 2. What has led to this situation? What has happened before? 3. What are the people thinking about, feeling, or wanting? 4. What will happen next? How will everything turn out? For each question one minute was given. After four minutes the subjects could correct their answers for a further minute.

The stories were coded with the Heckhausen coding system. This coding system consists of five main categories for hope of success (HS) and six main categories for fear of failure (FF) and one weighting category for each. The main categories for HS are: expression of the need for achievement and success (NS), instrumental activity to achieve success (IS), expectation of success (ES), praise (P) and positive affect (A+). For FF the main categories are need to avoid a failure (NF) and instrumental activity to avoid failure (IF), expectation of failure (EF), negative affect (A-), criticism (C) and failure (F). When the story of a picture fits the criteria of a category, one point was given, otherwise a score of 0. For the picture-scores the points were summed up. An additional point was given, when a story is primarily “success-seeking” (ST) or “failure-avoiding” (FT). The success theme is given when NS or ES are scored and no failure category excepting A- and EF. The failure theme is given when NF and F are scored and no success category excepting IS ([6]).

### Analysis

For testing our hypothesis we calculated Guttman’s λ_1_ to λ_6_ with SPSS and McDonald’s ω_t_ with the R psych packages for both, category and picture-scores, and the dichotomous data [36,37]. As the psych package uses correlation instead of covariances, which do not conform exactly to our equations. But before analysing we proofed the inter-rater-agreement of each two trained coders assessed with the *a*_*d*_-coefficient [38] and Pearson correlations (given in brackets). In our data set *a*_*d*_ was .998 for HS (*r* = .90) and .998 for FF (*r* = .87), which is in both cases above the 95 % level. The inter-rater-agreement for the data of [35] is also very well: *a*_*d*_ was .999 for HS (*r* = .96) and .999 for FF (*r* = .97).

## Results

Table 3 and 4 reveal the striking finding that using categories instead of pictures as items leads to higher scores. HS reliability measures using pictures as items were α = .22 (.12 in the student sample and .47 in the pupil sample) but with categories as items α increased to .48 (.52 in the student sample and .67 in the pupil sample). The same increase was found for FF, especially in the origin data sample of Heckhausen using categories instead of pictures which lead to an increase from a negative α (-.02) to .60. Moreover, the λ_5_ reliability coefficient for HS calculated using categories was .61 (in both samples, .68 in the pupil sample), which was higher than the coefficients using pictures (.36 in the Heckhausen sample, .22 in the student-sample and .50 in Pupil sample). The same preference for categories was found for FF. λ_5_ calculated for pictures was .20 in both investigations (.40 in the pupil sample), which was lower than the coefficients when calculating it for categories (.65 in the Heckhausen sample, .51 in the student sample, and .71 in the pupil sample). Regardless of the score used, the calculated reliability coefficients of λ_2_ and ω_t_ for pictures never outperformed the coefficients calculated using categories. When having a look on the reliability scores without considering the weighting categories (ST and FT, given for stories which fitting the motive very well), we still found that the reliability coefficients calculated using categories to be higher to those using pictures (e.g. .43 for FF in the Heckhausen sample calculated by categories vs. -.05 calculated by pictures). Generally the values of the coefficients for the setting without ST and FT are mostly lower but especially for the student sample some scores are even higher.

**Table 3:** Reliability-coefficients [15,22] or categories and pictures regarding the two scales hope for success and fear of failure with weighting categories and without (below).

	Hope of Success			Fear of Failure	
λ	Category	Picture	Category		Picture
1	.40/.44/.59	.18/.10/.40	.51/.27/.58		-.02/-.10/.30
2	.59/.59/.69	.36/.22/.49	.65/.38/.71		.17/.18/.39
3 = α	.48/.52/.67	.22/.12/.47	.60/.31/.68		-.02/.10/.36
4	.62/.52/.76	.28/.16/.46	.55/.37/.67		-.55/-.55/.33
5	.61/.61/.68	.36/.22/.50	.65/.51/.71		.20/.20/.40
6	.69/.57/.69	.35/.17/.45	.66/.37/.74		.16/.16/.35
ω_t_	.67/.54/.84	.54/.41/.64	.69/.42/.79		.47/.28/.42
Items	6	6	7		6

Note. The first of the three coefficients listed for each λ is from the Heckhausen data set (N = 35); the second coefficient is from the study with students (N = 113); and the third coefficient is from the pupil sample (N = 241).

**Table 4:** Reliability-coefficients [15,22] for categories and pictures regarding the two scales hope for success and fear of failure without weighting categories.

	Hope of Success		Fear of Failure	
λ	Category	Picture	Category	Picture
1	.09/.46/.30	.06/.33/.10	.36/.51/.07	-.05/.16/.07
2	.26/.60/.44	.24/.46/.21	.50/.64/.19	.16/.34/.17
3 = α	.11/.57/.37	.07/.40/.12	.43/.61/.08	-.05/.19/.08
4	-.09/.59/.23	.21/.50/.11	.21/.47/.17	-.65/-.38/-.01
5	.27/.61/.47	.25/.45/.21	.50/.66/.21	.19/.35/.18
6	.22/.55/.39	.21/.44/.16	.48/.61/.15	.14/.32/.14
ω_t_	.47/.32 /.63	.50/.37/.42	.59/.39 /.61	.49/.36/.63
Items	5	6	6	6

Note. The first of the three coefficients listed for each λ is from the Heckhausen data set (N = 35); the second coefficient is from the study with students (N = 113); and the third coefficient is from the pupil sample (N = 241).

To prove if the higher values of internal consistency result from the higher intercorrelations of the categories the intercorrelations for the Heckhausen data is given in table 5 and table 6.

**Table 5:** Intercorrelations of pictures-scores.

	A	B	C	D	E	F	*M*	*SD*	*r_it_*
A		-.25	.27	.09	-.26	.27	2.74	1.27	.31
B	-.10		-.01	-.06	-.04	-.30	0.11	0.32	.31
C	.04	.03		.42*	.00	.28	2.00	1.55	.20
D	-.08	-.13	.11		-.05	.10	0.06	0.24	.23
E	-.13	.01	.05	.28		-.03	1.74	1.27	.36
F	.07	-.27	-.35*	.22	.35*		0.29	0.62	-.23
*M*	0.14	2.20	0.26	2.00	0.40	0.89			
*SD*	0.36	1.45	0.61	1.68	0.91	1.08			
*r_it_*	.16	-.08	-.05	.20	.25	-.17			

Note. Correlation coefficients over the diagonal refer to HS, below refer to FF, Heckhausen data set n = 35, * p < .05

**Table 6:** Intercorrelations of category-scores.

	NS/NF	IS/IF	ES/EF	P/C	A+/A-	/F	ST/FT	*M*	*SD*	*r_it_*
NS/NF		.08	-.11	-.04	-.36*		.64**	1.09	0.95	.67
IS/IF	-.05		.26	.00	.06		.36*	2.46	0.78	.57
ES/EF	.07	-.06		-.13	.34*		.35*	0.49	0.66	.16
P/C	.22	-.21	.28		.12		-.13	0.20	0.47	.02
A+/A-	.08	.01	.20	.44**			.20	1.26	0.92	.08
/F	.03	.02	.06	.44**	.40*		-	1.46	1.17	.55
ST/FT	.43**	.02	.11	.41*	.56**	.53**		1.09	0.95	.66
*M*	0.43	0.57	1.14	0.23	1.91	0.77	0.83			
*SD*	0.61	0.98	0.97	0.43	1.04	0.84	0.89			
*r_it_*	.30	.18	.15	.12	.25	.00	.42			

Note. Correlation coefficients over the diagonal refer to HS, below refer to FF, Heckhausen data set n = 35, * p < .05, ** p < .01

For HS (above the diagonal) the correlations of the categories are not as clearly higher as expected compared with the correlations of the picture-scores. But for FF it can be observed. On the other hand the mean (via Fisher-transformation) correlation of .03 for the HS picture-scores is clearly lower than the mean correlation of .12 for the HS category-scores. As the mean FF picture-scores correlation is .00, the mean correlation of the FF category-scores is .20. Similar results can be observed for the two other data sets.

The values of the reliability coefficients calculated on a dichotomous level are similar to the values observed for category-scores (see table 7). On this dichotomous level ω_t_ should be able to be calculated using a standard algorithm as an approximation. But for an exact assessment nonlinear factor models are required ([22], p. 102f). Both options were not available in all R-packages that we reviewed [36,37].

**Table 7:** Reliability-coefficients regarding to the two scales hope for success and fear of failure with dichotomous data for categories-by-pictures.

	Hope of Success		Fear of Failure
λ_3_ (resp. KR-20)	.50 / .56 /.70	.52 / .42 /.68	
Items	36	42	

Note. The first of the three coefficients listed is from the Heckhausen data set (N = 35); the second coefficient is from the study with students (N = 113); and the third coefficient is from the pupil sample (N = 241).

We expected that the reliability estimated with dichotomous data would be influenced by the pictures score and the categories score reliability. Thus, this value was expected to be between category and picture reliability. The sample of Heckhausen confirmed our assumption for FF (α: .60 > .52 > -.02) but not for HS. In contrast, the pupil sample confirmed the assumption for HS (α: .76 > .70 > -.47) but not for FF. Neither pattern was found in the sample of students for HS or FF.

## Conclusion

Calculating reliability of PSE has long been noted as a persistent problem, which we contend has been independent of the test: The problem was the result of treating this method as a self-report-measurement, but Picture Stories Exercises are different. The underlying phenomena, explained in the Dynamics of Action theory as saw-tooth-effect and in the Cognitive Affective System Theory as picture-cue-effect, decrease the homogeneity of items. This effects, however, does not negatively impact the coding system. Investigating the reliability of the tests on the basis of the coding system can provide a solution. We found evidence to confirm this hypothesis in three different data sets. On the one hand there are clear higher intercorrelations when the category-scores used as items compared to the picture-scores (table 5, table 6) and on the other most of and especially the preferred coefficients for internal consistency λ_2_ and ω_t_ are higher for category-scores. In future studies the hypothesized relationship between reliability calculated using pictures or categories should be assessed with Monte Carlo simulation to confirm our theoretical assumptions and further demonstrate the superiority of calculating reliability coefficients using coding categories instead of pictures.

We strongly advise to refrain from using the α coefficient on the basis of picture-scores because of two main reasons. First, pictures are compromised by the saw-tooth-effect and/or the picture-cue-effect. Second, α is an appropriate measure for homogenous data, but not for projective tests such as PSE. λ_2_, λ_5_ and ω_t_ are more appropriate measures, because they better fit the theoretical concept of projective tests. We also dissuade from using Rasch-scaling for dichotomous data to estimate PSE reliability, because the prerequisites of stochastic independence cannot be fulfilled and the procedure does not fit the theoretical concept of PSE. On the other hand, the item response theory for ordinal data (for the category-scores) could be worth to examine in further research as a possible adequate measurement model for PSE and projective tests.The results of our study are limited to the PSE and the coding system of Heckhausen [3]. Regarding the possible dissent that categorical reliability is only higher because of the weighting categories we have shown that in both conditions, with and without weighting categories, categorical reliability always outperforms pictorial reliability. For the weighting categories do not only depend on the positive categories but also on the absence of negative categories, it is not just a lifting effect as the results for the student sample accessorily clarified. Further research is needed to replicate the effects on different projective tests, different coding systems, in different countries, and both clinical and nonclinical groups. Our method can also be adapted to other verbal-thematic projective tests for which stories or statements are produced in response to a picture and then coded by a categorical system. For example, the Fairy-Tale Test [39] and the Rosenzweig Picture-Frustration Test [40] and all modifications of TAT and PSE based on a categorical system are possible. Applying the method to sentence- and story-completing tests and drawing tests would also be appropriate, when there is a categorical system. Researchers using these tests could benefit from our method; hence further investigations are needed in this area.

## Supporting Information

Appendix S1 **Mathematical Proofs, AppendixS1.**
(PDF)

Data Set S1 **Heckhausen data set.**
(DAT)

Data Set S2 **Breidebach data set.**
(DAT)

Data Set S3 **Students data set.**
(DAT)

Figure S1 **Variance-covariance-matrix for two pictures and two categories, Figure S1.**
(TIF)

Figure S2 **Variance-covariance-matrix for total TAT-ratings with pictures from A to F and categories from 1 to 6, Figure S2.**
(TIF)
