# Supplementary material for: Measuring the Reliability of Picture Story Exercises like the TAT
Source: PLoS One. 2013 Nov 5;8(11):e79450. doi: 10.1371/journal.pone.0079450 (PMC3865338; doi:10.1371/journal.pone.0079450)
Supplement: Appendix S1 — Mathematical Proofs, AppendixS1. (PDF) [file pone.0079450.s001.pdf]

Appendix S1: Mathematical Proofs

The variance of a scale is defined as the squared sum of each score subtracted from the mean of the score divided by *n*:

$$Var_j = \frac{1}{n}\sum_{j=1}^{N}(x_j - \bar{x}_j)^2$$

If a test consist of two pictures (p_1, p_2) and two categories (c_1, c_2), the matrix of all possible covariances can depict as in figure S2.

As obvious in figure S2 the total sum of all covariances is expressed as $Cov_c + Cov_p + Cov_{pc} + Var_{pc}$.

The sum of all subvariances (the variances of all subitems x_{pc}) is similar to the diagonal of the variance matrix ([14] p. 303) and can be expressed as followed:

$$Var_{pc} = \frac{1}{n}\left[\sum\left(x_{j_{p1c1}} - \bar{x}_{j_{p1c1}}\right)^2 + \sum\left(x_{j_{p1c2}} - \bar{x}_{j_{p1c2}}\right)^2 + \sum\left(x_{j_{p2c1}} - \bar{x}_{j_{p2c1}}\right)^2 + \sum\left(x_{j_{p2c2}} - \bar{x}_{j_{p2c2}}\right)^2\right]$$

for j indicates counting up from first to last subject of the test.

The sum of covariances of categories will be the covariance of category one and two, which can be written as:

$$2 \cdot Cov_c = \frac{2}{n}\left[\sum\left(x_{j_{p1c1}} - \bar{x}_{j_{p1c1}}\right)\left(x_{j_{p1c2}} - \bar{x}_{j_{p1c2}}\right) + \sum\left(x_{j_{p2c1}} - \bar{x}_{j_{p2c1}}\right)\left(x_{j_{p2c2}} - \bar{x}_{j_{p2c2}}\right)\right]$$

The sum of covariances of pictures will be the covariance of picture one and two which can be written as:

$$2 \cdot Cov_p = \frac{2}{n}\left[\sum\left(x_{j_{p1c1}} - \bar{x}_{j_{p1c1}}\right)\left(x_{j_{p2c1}} - \bar{x}_{j_{p2c1}}\right) + \sum\left(x_{j_{p1c2}} - \bar{x}_{j_{p1c2}}\right)\left(x_{j_{p2c2}} - \bar{x}_{j_{p2c2}}\right)\right]$$

The next step demonstrates mathematically that this Formula truly represents the covariances, and that the sum $Var_{pc} + 2Cov_c + 2Cov_p + 2Cov_{pc}$ is the total test variance. Figure S3 gives a detailed view of the total covariance-variance of an exemplary TAT-Picture-Category-Matrix.

As obvious above the total test variance expresses the mean squared deviation of the mean. For n is constant just the sum of squares (SS_t) are taken into account:

$$SS_t = \sum_{j=1}^{N}(x_j - \bar{x}_j)^2; \quad SS_t = \sum_{j=1}^{N}(x_j{}^2 - 2(x_j\bar{x}_j) + \bar{x}_j{}^2)\ ; \quad x_{jpc} = \sum_{p=1}^{P}\sum_{c=1}^{C}(x_{jpc})$$

$$SS_t = \sum_{j=1}^{N}\left(\sum_{p=1}^{P}\sum_{c=1}^{C}x_{jpc}\right)^2 - 2\left[\left(\sum_{p=1}^{P}\sum_{c=1}^{C}x_{jpc}\right)\left(\sum_{p=1}^{P}\sum_{c=1}^{C}\bar{x}_{jpc}\right)\right] + \left(\sum_{p=1}^{P}\sum_{c=1}^{C}\bar{x}_{jpc}\right)^2$$

Solving this equation with the theorem for squared sums

$$\left(\sum_{c=1}^{C}x_c\right)^2 = \sum_{c=1}^{C}(x_c)^2 + 2\sum_{c=1}^{C-1}\sum_{b=c+1}^{C}(x_c x_b)$$

leads to:

$$SS_t = \sum_{j=1}^{N}\left[\left(\sum_{p=1}^{P}\sum_{c=1}^{C}(x_{jpc})^2\right) + 2\left(\sum_{p=1}^{P-1}\sum_{a=p+1}^{P}\sum_{c=1}^{C}x_{jpc}x_{jac}\right) + 2\left(\sum_{p=1}^{P}\sum_{c=1}^{C-1}\sum_{b=c+1}^{C}x_{jpc}x_{jpb}\right) + 2\left(\sum_{p=1}^{P-1}\sum_{a=p+1}^{P}\sum_{c=1}^{C-1}\sum_{b=c+1}^{C}x_{jpc}x_{jab}\right)\right]$$

$$-\sum_{j=1}^{N}\left[2\left(\sum_{p=1}^{P}\sum_{c=1}^{C}(x_{jpc}\bar{x}_{jpc})\right) + 2\left(\sum_{p=1}^{P-1}\sum_{a=p+1}^{P}\sum_{c=1}^{C}x_{jpc}\bar{x}_{jac}\right) + 2\left(\sum_{p=1}^{P}\sum_{c=1}^{C-1}\sum_{b=c+1}^{C}x_{jpc}\bar{x}_{jpb}\right) + 2\left(\sum_{p=1}^{P-1}\sum_{a=p+1}^{P}\sum_{c=1}^{C-1}\sum_{b=c+1}^{C}x_{jpc}\bar{x}_{jab}\right)\right]$$

$$-\sum_{j=1}^{N}\left[2\left(\sum_{p=1}^{P-1}\sum_{a=p+1}^{P}\sum_{c=1}^{C}x_{jac}\bar{x}_{jpc}\right) + 2\left(\sum_{p=1}^{P-1}\sum_{c=1}^{C-1}\sum_{b=c+1}^{C}x_{jpb}\bar{x}_{jpc}\right) + 2\left(\sum_{p=1}^{P-1}\sum_{a=p+1}^{P}\sum_{c=1}^{C-1}\sum_{b=c+1}^{C}x_{jab}\bar{x}_{jpc}\right)\right]$$

$$+\sum_{j=1}^{N}\left[\left(\sum_{p=1}^{P}\sum_{c=1}^{C}(\bar{x}_{jpc})^2\right) + 2\left(\sum_{p=1}^{P-1}\sum_{a=p+1}^{P}\sum_{c=1}^{C}\bar{x}_{jpc}\bar{x}_{jac}\right) + 2\left(\sum_{p=1}^{P}\sum_{c=1}^{C-1}\sum_{b=c+1}^{C}\bar{x}_{jpc}\bar{x}_{jpb}\right) + 2\left(\sum_{p=1}^{P-1}\sum_{a=p+1}^{P}\sum_{c=1}^{C-1}\sum_{b=c+1}^{C}\bar{x}_{jpc}\bar{x}_{jab}\right)\right]$$

$$SS_t = \sum_{j=1}^{N}\sum_{p=1}^{P}\sum_{c=1}^{C}x_{jpc}^2 - 2\sum_{j=1}^{N}\sum_{p=1}^{P}\sum_{c=1}^{C}(x_{jpc}\bar{x}_{jpc}) + \sum_{j=1}^{N}\sum_{p=1}^{P}\sum_{c=1}^{C}\bar{x}_{pc}^2$$

$$+2\left[\sum_{j=1}^{N}\sum_{p=1}^{P-1}\sum_{a=p+1}^{P}\sum_{c=1}^{C}(x_{jpc}x_{jac}) - \sum_{j=1}^{N}\sum_{p=1}^{P-1}\sum_{a=p+1}^{P}\sum_{c=1}^{C}(x_{jpc}\bar{x}_{jac}) - \sum_{j=1}^{N}\sum_{p=1}^{P-1}\sum_{a=p+1}^{P}\sum_{c=1}^{C}(\bar{x}_{jpc}x_{jac}) + \sum_{j=1}^{N}\sum_{p=1}^{P-1}\sum_{a=p+1}^{P}\sum_{c=1}^{C}(\bar{x}_{jpc}\bar{x}_{jac})\right]$$

$$+2\left[\sum_{j=1}^{N}\sum_{p=1}^{P}\sum_{c=1}^{C-1}\sum_{b=c+1}^{C}(x_{jpc}x_{jpb}) - \sum_{j=1}^{N}\sum_{p=1}^{P}\sum_{c=1}^{C-1}\sum_{b=c+1}^{C}(x_{jpc}\bar{x}_{jpb}) - \sum_{j=1}^{N}\sum_{p=1}^{P}\sum_{c=1}^{C-1}\sum_{b=c+1}^{C}(\bar{x}_{jpc}x_{jpb}) + \sum_{j=1}^{N}\sum_{p=1}^{P}\sum_{c=1}^{C-1}\sum_{b=c+1}^{C}(\bar{x}_{jpc}\bar{x}_{jpb})\right]$$

$$+2\left[\sum_{j=1}^{N}\sum_{p=1}^{P-1}\sum_{a=p+1}^{P}\sum_{c=1}^{C-1}\sum_{b=c+1}^{C}(x_{jpc}x_{jac}) - \sum_{j=1}^{N}\sum_{p=1}^{P-1}\sum_{a=p+1}^{P}\sum_{c=1}^{C-1}\sum_{b=c+1}^{C}(x_{jpc}\bar{x}_{jac}) - \sum_{j=1}^{N}\sum_{p=1}^{P-1}\sum_{a=p+1}^{P}\sum_{c=1}^{C-1}\sum_{b=c+1}^{C}(\bar{x}_{jpc}x_{jac}) + \sum_{j=1}^{N}\sum_{p=1}^{P-1}\sum_{a=p+1}^{P}\sum_{c=1}^{C-1}\sum_{b=c+1}^{C}(\bar{x}_{jpc}\bar{x}_{jac})\right]$$

$$SS_t = \sum_{j=1}^{N}\sum_{p=1}^{P}\sum_{c=1}^{C}(x_{jpc} - \bar{x}_{jpc})^2 + 2\sum_{j=1}^{N}\sum_{p=1}^{P-1}\sum_{a=p+1}^{P}\sum_{c=1}^{C}\left[(x_{jpc} - \bar{x}_{jpc})(x_{jac} - \bar{x}_{jac})\right]$$

$$+2\sum_{j=1}^{N}\sum_{p=1}^{P}\sum_{c=1}^{C-1}\sum_{b=c+1}^{C}\left[(x_{jpc} - \bar{x}_{jpc})(x_{jpb} - \bar{x}_{jpb})\right] + 2\sum_{j=1}^{N}\sum_{p=1}^{P-1}\sum_{a=p+1}^{P}\sum_{c=1}^{C-1}\sum_{b=c+1}^{C}\left[(x_{jpc} - \bar{x}_{pc})(x_{jab} - \bar{x}_{jab})\right]$$

These components are exactly the sum of square of variances (v), pictural covariance (p), categorical covariances (c) and general covariances (g), as so coloured in figure S2. But this equation also shows that the overall variance can be calculated as a sum of pictures variances

and twice their covariances or category variances and twice their covariances. Let SS_p be the sum of squares for picture variance and SS_c the sum of square for category variance, and SS'_p the covariance multiplied with n for picture (SS'_p) and category (SS'_c):

$$SS_p = \sum_{j=1}^{N}(x_{jp} - \bar{x}_{jp})^2; \; for\ x_{jpc} = \sum_{p=1}^{P}\sum_{c=1}^{C}(x_{jpc}); \quad SS_p = \sum_{j=1}^{N}\sum_{p=1}^{P}\left[\sum_{c=1}^{C}(x_{jpc} - \bar{x}_{jpc})\right]^2$$

$$SS_p = \sum_{j=1}^{N}\sum_{p=1}^{P}\sum_{c=1}^{C}(x_{jpc} - \bar{x}_{jpc})^2 + 2\sum_{j=1}^{N}\sum_{p=1}^{P}\sum_{c=1}^{C-1}\sum_{b=c+1}^{C}\left[(x_{jpc} - \bar{x}_{jpc})(x_{jpb} - \bar{x}_{jpb})\right]$$

$$SS_c = \sum_{j=1}^{N}(x_{jc} - \bar{x}_{jc})^2; \; for\ x_{jpc} = \sum_{p=1}^{P}\sum_{c=1}^{C}(x_{jpc}); \quad SS_c = \sum_{j=1}^{N}\sum_{c=1}^{C}\left[\sum_{p=1}^{P}(x_{jpc} - \bar{x}_{jpc})\right]^2 =$$

$$SS_c = \sum_{j=1}^{N}\sum_{p=1}^{P}\sum_{c=1}^{C}(x_{jpc} - \bar{x}_{jpc})^2 + 2\sum_{j=1}^{N}\sum_{p=1}^{P-1}\sum_{a=p+1}^{P}\sum_{b=c+1}^{C}\left[(x_{jpc} - \bar{x}_{jpc})(x_{jac} - \bar{x}_{jac})\right]$$

$$SS'_p = \sum_{j=1}^{N}\sum_{p=1}^{P-1}(x_{jp} - \bar{x}_{jp})(y_{jp} - \bar{y}_{jp}) \quad \text{for } y \neq x \text{ leads to } SS'_p = \sum_{j=1}^{N}\sum_{p=1}^{P-1}\sum_{a=p+1}^{P}(x_{jp} - \bar{x}_{jp})(x_{ja} - \bar{x}_{ja}) \quad \text{for } x_j = \sum_{c=1}^{C}x_{jc}$$

$$SS'_p = \sum_{j=1}^{N}\sum_{p=1}^{P-1}\sum_{a=p+1}^{P}\left[\sum_{c=1}^{C}(x_{jpc} - \bar{x}_{jpc})\sum_{c=1}^{C}(x_{jac} - \bar{x}_{jac})\right]$$

$$SS'_p = \sum_{j=1}^{N}\sum_{p=1}^{P-1}\sum_{a=p+1}^{P}\left[\sum_{c=1}^{C}(x_{jpc} - \bar{x}_{jpc})(x_{jac} - \bar{x}_{jac}) + \sum_{c=1}^{C-1}\sum_{b=c+1}^{C}(x_{jpc} - \bar{x}_{jpc})(x_{jab} - \bar{x}_{jab})\right]$$

$$SS'_p = \sum_{j=1}^{N}\sum_{p=1}^{P-1}\sum_{a=p+1}^{P}\sum_{c=1}^{C-1}\sum_{b=c+1}^{C}\left[(x_{jpc} - \bar{x}_{jpc})(x_{jac} - \bar{x}_{jac})\right] + \sum_{j=1}^{N}\sum_{p=1}^{P-1}\sum_{a=p+1}^{P}\sum_{c=1}^{C-1}\sum_{b=c+1}^{C}\left[(x_{jpc} - \bar{x}_{jpc})(x_{jab} - \bar{x}_{jab})\right]$$

$$SS'_c = \sum_{j=1}^{N}\sum_{c=1}^{C-1}\sum_{b=c+1}^{C}\left[\sum_{p=1}^{P}(x_{jpc} - \bar{x}_{jpc})\sum_{p=1}^{P}(x_{jpb} - \bar{x}_{jpb})\right]$$

$$SS'_c = \sum_{j=1}^{N}\sum_{c=1}^{C-1}\sum_{b=c+1}^{C}\left[\sum_{p=1}^{P}(x_{jpc} - \bar{x}_{jpc})(x_{jpb} - \bar{x}_{jpb}) + \sum_{p=1}^{P-1}\sum_{a=p+1}^{P}(x_{jpc} - \bar{x}_{jpc})(x_{jab} - \bar{x}_{jab})\right]$$

$$SS'_c = \sum_{j=1}^{N}\sum_{p=1}^{P-1}\sum_{a=p+1}^{P}\sum_{c=1}^{C-1}\sum_{b=c+1}^{C}\left[(x_{jpc} - \bar{x}_{jpc})(x_{jpb} - \bar{x}_{jpb})\right] + \sum_{j=1}^{N}\sum_{p=1}^{P-1}\sum_{a=p+1}^{P}\sum_{c=1}^{C-1}\sum_{b=c+1}^{C}\left[(x_{jpc} - \bar{x}_{jpc})(x_{jab} - \bar{x}_{jab})\right]$$

Now taking these similarities and differences of SS'_p and SS'_c into account for the calculation of α using the category-scores and the picture-scores (see also Eq. 3a and 3b):

Into

$$\alpha = \frac{n}{(n-1)} \cdot \frac{2Ct}{Vt} \Leftrightarrow n \cdot Ct = 0.5\,(n-1)\,Vt\,\alpha$$

for categories the decomposition of C_c into the blue term (c) and the yellow term (g, see above) and for pictures into the green term (p) and the yellow term (g) as well will inserted:

$n \cdot C_c = c + g$ and $n \cdot C_p = p + g$

$c + g = 0.5\ (n-1)\ Vt\ \alpha_c$ and $p + g = 0.5\ (n-1)\ Vt\ \alpha_p$

Resolving both sides to g and equate to each other leads to the equation of α_c as function of α_p:

$0.5\ (n-1)\ Vt\ \alpha_c\ \text{-}\ c = 0.5\ (n-1)\ Vt\ \alpha_p\ \text{-}\ p$

$$\Leftrightarrow\ \alpha_c = \frac{0.5\ (n\text{-}1)\ Vt\ \alpha_p\ \text{-}p+c}{0.5\ (n\text{-}1)\ Vt} = \alpha_p + \frac{2\ (c-p)}{(n\text{-}1)\ Vt} \qquad \text{(S1)}$$
