# Supplementary figures and images for: Measuring the Reliability of Picture Story Exercises like the TAT
Source: PLoS One. 2013 Nov 5;8(11):e79450. doi: 10.1371/journal.pone.0079450 (PMC3865338; doi:10.1371/journal.pone.0079450)

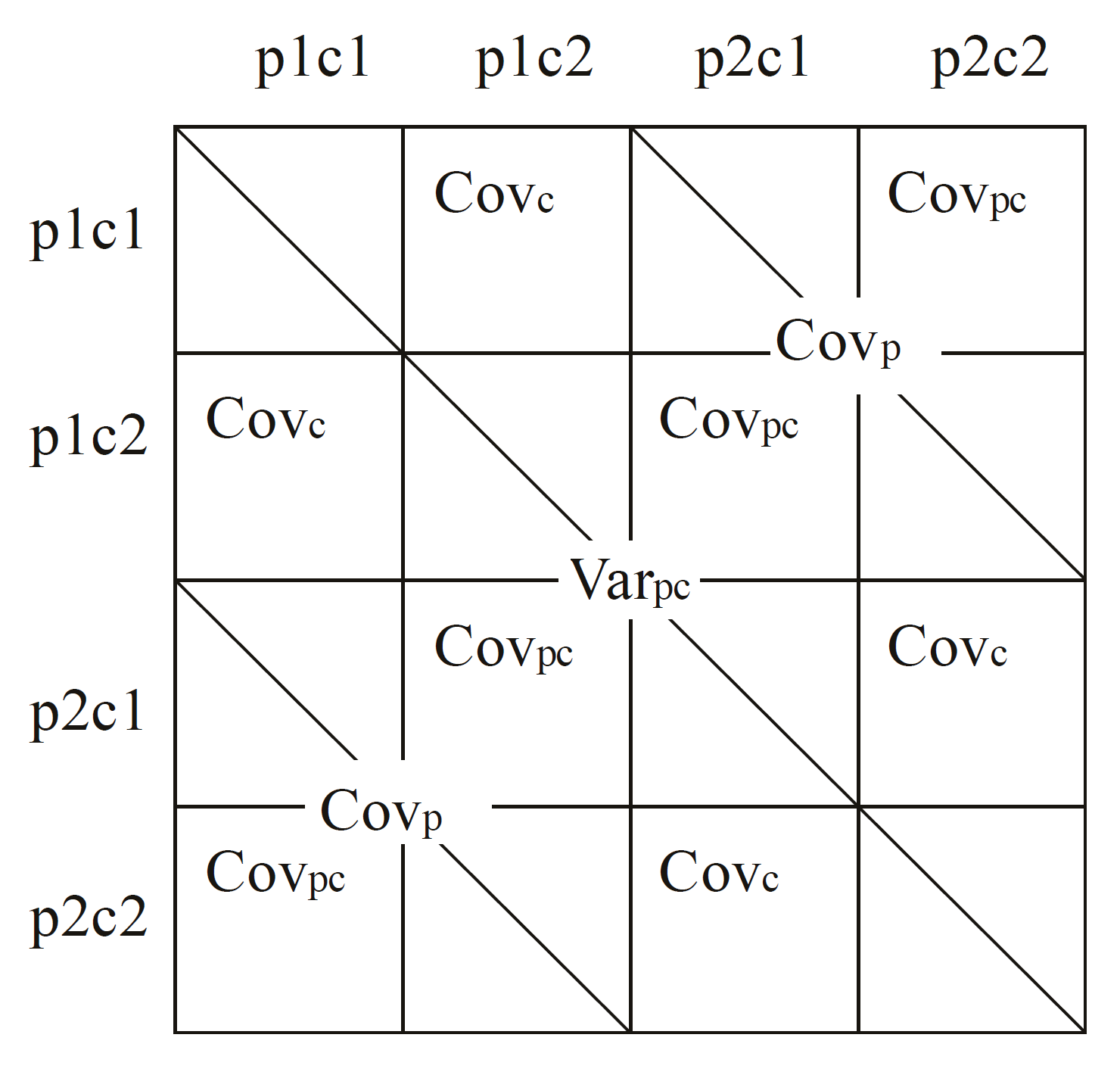

Supplement: Figure S1 — Variance-covariance-matrix for two pictures and two categories, Figure S1. (TIF) [file pone.0079450.s005.tif]

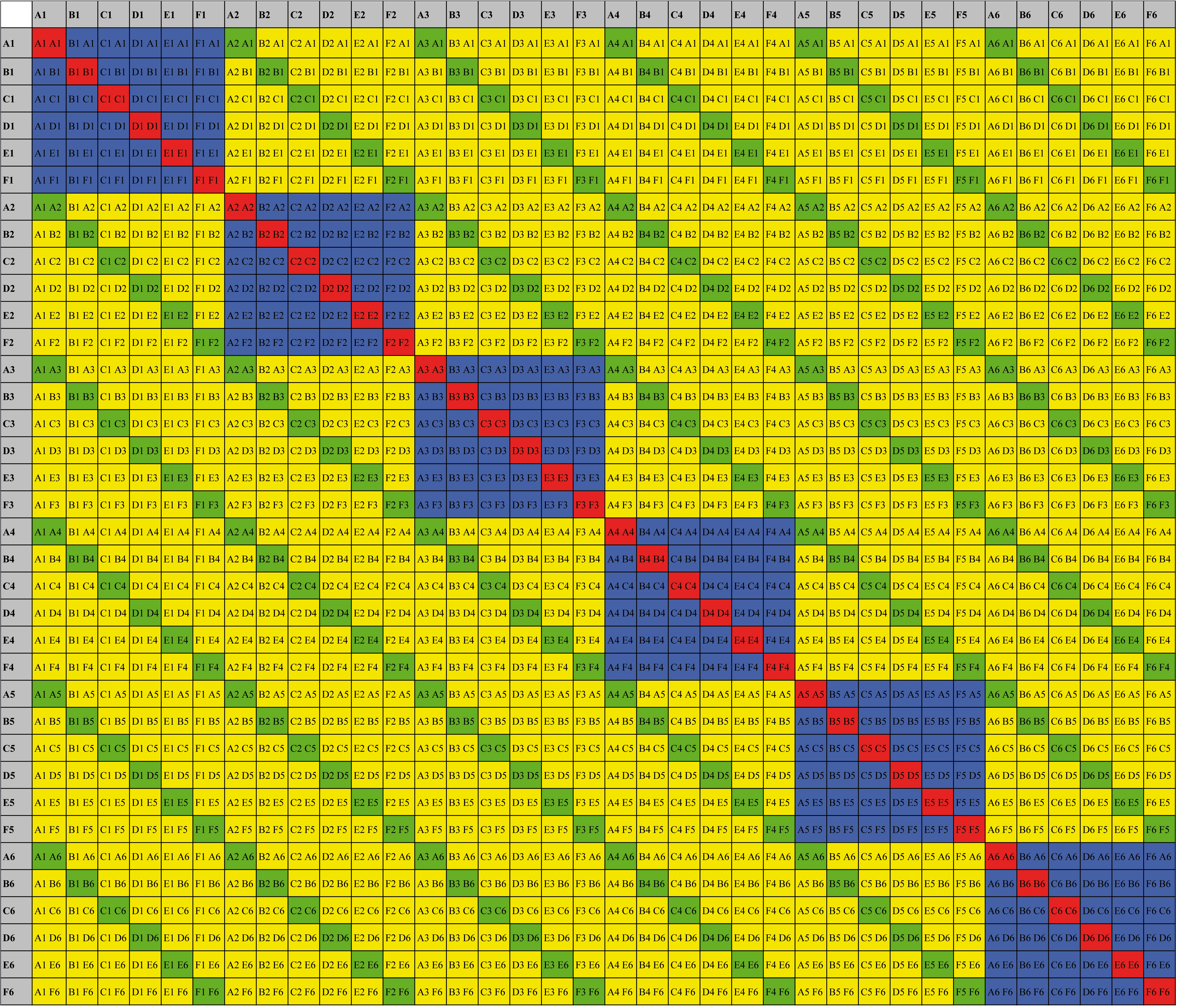

Supplement: Figure S2 — Variance-covariance-matrix for total TAT-ratings with pictures from A to F and categories from 1 to 6, Figure S2. (TIF) [file pone.0079450.s006.tif]
